# Orthorexia Nervosa, Eating Disorders and Obsessive-Compulsive Disorder: A Selective Review of the Last Seven Years

**DOI:** 10.1192/j.eurpsy.2025.1410

**Published:** 2025-08-26

**Authors:** M. A. Rodrigues, F. M. Amorim, M. H. R. Delgado, L. A. Lemes

**Affiliations:** 1 Universidade Anhembi Morumbi, São Paulo, Brazil

## Abstract

**Introduction:**

Orthorexia Nervosa (ON) is an eating disorder, characterized by rigid and irrational rules about nutrition and food preparation. Although it is not officially recognized by the DSM-5 as a mental disorder, individuals with ON exhibit compulsive and obsessive behaviors related to their diets, with an interest that starts moderate but gradually becomes excessive and potentially pathological.

**Objectives:**

To assess individuals facing eating disorders, with an emphasis on Orthorexia Nervosa (ON) and Obsessive-Compulsive Disorder (OCD).

**Methods:**

This is an integrative literature review, with analyses conducted on the National Library of Medicine (PUBMED) using the descriptors and boolean operators: (Orthorexia Nervosa) AND (Eating Disorders) AND (Obsessive-Compulsive Disorder) AND (obsessive compulsive). Filters applied included: articles published in the last 10 years and available in full text. All articles were analyzed a priori based on the information in the title, followed by reading the abstract, and subsequently, reading the full article.

**Results:**

Seventeen studies were found. According to the data, the age range between 23 and 26 years is at the highest risk for ON, while the risk is lower in individuals between 16 and 19 years. Orthorexic tendencies are more prevalent among Hispanics/Latinos and individuals with overweight or obesity. ON is frequently associated with patients who have body distortions, dissatisfaction with body image, excessive desire for thinness, and eating disorders such as anorexia and bulimia nervosa. Additionally, ON is linked to compulsive behaviors, obesity, strict diets, perfectionism. In individuals on diets, behaviors and problems similar to those seen in eating disorders are often observed, including orthorexic tendencies and obsession with adhering to strict dietary rules. Perfectionism is a common trait among these patients, who often begin with the desire to improve their diet and adopt a healthier lifestyle. However, this pursuit can become excessive, leading to behaviors such as social isolation, high spending on food, and nutritional deficiencies. Various tests conducted in the analyzed studies showed effects in identifying patients with ON, indicating the need for further research to better understand risk factors in different populations and to develop effective strategies for diagnosis, prevention, and treatment.

**Conclusions:**

While a careful diet is not pathological in itself, severe dietary restrictions and social problems should raise the possibility of ON. Improving the identification of risk factors and behaviors may contribute to a better understanding, prevention, and management of ON.

**Disclosure of Interest:**

None Declared

